# Growth Performance of Sandalwood (*Santalum album*) Plant in Western Nepal

**DOI:** 10.1155/tswj/3052342

**Published:** 2025-02-15

**Authors:** Manju Paudel, Gyan Bandhu Sharma, Gandhiv Kafle

**Affiliations:** Faculty of Forestry, Agriculture and Forestry University, Hetauda, Nepal

**Keywords:** diameter, height, sandalwood, volume increment

## Abstract

Sandalwood is one of the exotic species of Nepal. Among all, Sandalwood (*Santalum album*) is found cultivated in a private land by local farmers. Sandalwood oil is used in perfumes, cosmetics, aromatherapy, and pharmaceuticals. The research was carried out to assess the growth performance of Sandalwood in particular soil condition in the Pyuthan district of Nepal. Four villages of Swargadwari municipality, Ward No. 3 of the Pyuthan district where large numbers of Sandalwood plants have been planted by local households on their private land, were selected. The objective of the study was to document the growth performance of sandalwood in terms of mean annual volume increment. Data collection was carried out through primary and secondary data sources. Primary data were collected through the diameter and height measurement of the Sandalwood tree by using a diameter tape, Abney's level, and a linear tape. As supportive data source, secondary data were collected from different journal articles, yearly publications of the Division Forest Office, Pyuthan, Department of Forest and Soil Conservation, and Ministry of Forest and Environment. The collected data were analyzed using descriptive statistics. The results were presented in the form of a map, table, and bar graph. The highest mean annual height increment was 0.51 m/year at the age of 10 years; similarly, the lowest mean annual height increment was 0.44 m/year at the age of 16 years. The highest mean annual diameter increment was 1.009 at the age of 15 years; similarly, the lowest mean annual diameter increment was 0.97 m/year at the age of years. The highest mean annual volume increment was 0.004 m^3^/year at the age of 15 years, and the lowest mean annual volume increment was 0.001 m^3^/year at the age of 9 years. The result shows a gradual increase in the mean annual volume increment with age from the age of 9–15 years, and it shows a slight reduction at the age of 16 years as compared to that of 15 years.

## 1. Introduction

*Santalum album* L., commonly known as Indian Sandalwood, is an evergreen, semiparasitic tree belonging to the Santalaceae family [1–3]. This species is renowned as one of the oldest and most valuable sources of natural fragrance, holding a significant therapeutic and commercial value [1, 4–6]. Sandalwood is globally esteemed for its distinctive, sweet aroma, long-lasting quality, and therapeutic properties. The oil extracted from the heartwood of Indian Sandalwood is utilized in traditional medical systems such as Ayurveda, Siddha, and Unani for treating and preventing various ailments [1, 7]. The value of the Indian Sandalwood tree primarily depends on the volume and quality of its heartwood and the essential oil it produces [1].

Indian Sandalwood (*S. album*) is predominantly found in East Asia, Australia, and South Asia, including India, Nepal, and Sri Lanka, particularly, at the Himalayan foothills. The tree is typically found at elevations up to 1200 m. Sandalwood holds a significant place in Indian culture and is considered the second most expensive wood globally. The heartwood of the tree is highly valued for its fragrance and is one of the finest natural materials for carving. Sandalwood oil is widely used in perfumes, cosmetics, aromatherapy, and medicinal preparations. Due to extensive exploitation driven by the monopolistic trade of Sandalwood, *S. album* is now listed as vulnerable on the IUCN Red List. Extensive research has highlighted the significant genetic diversity within this species [8].

In Nepal, *S. album* is considered an alien species. Local farmers often cultivate this type of sandalwood on their own property [9]. Globally, Indian Sandalwood (*S. album* L.) is highly valued for its heartwood and essential oil content, making it one of the most precious commercial timbers in the world [10]. It is recognized as the species within the Genus *Santalum* that produces the highest quality sandal oil among all sandalwood trees [11]. Although its growth rate can vary significantly, sandalwood is generally considered a slow-growing tree in its natural habitat. Under favorable conditions, it can grow 5-6 cm in girth annually. However, it grows more rapidly in plantation settings [12].

Pronk [13] highlights the pioneering research and commercial success of Indian Sandalwood plantations in Australia, which have become the world's largest plantation sandalwood supply. Similarly, the ACIAR report [14] discusses the domestication and breeding efforts of sandalwood in Fiji, aiming to establish a sustainable industry. Highlighting these examples will provide valuable context and demonstrate the adaptability of this species outside its native range [13, 14].

Additionally, the global market for sandalwood products remains strong, with a high demand for sandalwood oil and heartwood from various regions, including Australia and Fiji [15]. The economic potential of sandalwood cultivation has attracted significant investment and research efforts, further underscoring its adaptability and commercial viability [15].

To protect these trees from animals, which find them highly palatable, it is beneficial to use fence plants with prickly branches. Sandalwood is also extremely sensitive to fire. Regular weeding and pruning of host plants are essential to promote healthy growth in both the host and the sandalwood tree [16]. The literature suggests that combining sandalwood with horticultural species as secondary hosts can theoretically enhance its growth. Given the rising global market prices for sandalwood oil and wood, cultivating this tree can significantly support the local economy [10].

To meet the projected sustained demand, sandalwood must transition rapidly from being an overly regulated “forest product” to a commodity cultivated in plantation forestry. This transition involves numerous stages and thresholds, similar to those identified in the transitions of other forest products, both timber and nontimber. Three critical thresholds include domestication, intentional regeneration, and regulated consumption [17].

Sandalwood thrives in various soil types, including sand, clay, laterite, loam, and black-cotton soil, provided it avoids wet conditions. The best-quality, finest-smelling sandalwood is often found in the driest regions, especially on red or stony ground [18–20]. Optimal soil for sandalwood development includes rich, fairly moist soil like garden loam and well-drained deep alluvium on riverbanks. It requires adequate drainage and cannot tolerate waterlogged conditions [20]. As a slow-growing root parasite, sandalwood interacts with other components in a complex pattern of competition and host-parasite relationships, depending on the root distribution and rooting depth of a potential host [17].

Since the Vedic era, particularly during the rule of the Vijayanagara Empire and the Kings of Mysore, the heartwood of the sandalwood tree has been prized as the best material for carving, and its oil's fragrance has been valued globally by the perfumery industry [2, 21]. The output and quality of sandalwood oil are directly related to its market worth, making the accurate assessment of these factors crucial for international trade. Hydrodistillation is a popular method for measuring these characteristics and can be applied to larger commercial quantities of sandalwood. However, the plantation industry requires a more rapid and economical extraction method for smaller samples, such as individual heartwood cores from young trees.

Global natural sandalwood wealth is declining due to unsustainable harvesting from natural populations. This overexploitation has led to a substantial population decline and potential genetic impoverishment [22, 23].

With 500–3000 mm of rainfall, sandal trees may flourish on any kind of soil from sea level to 1800 m above sea level [24]. The best hardwood formations are thought to be found in dry areas with moderate temperatures and altitudes between 600 and 9 m.

Three main characters affecting the value of the Sandalwood tree are

i. The volume of heartwood
ii. The concentration of oil
iii. Quality of its heartwood oil [25, 26]

The development of heartwood in other tree species is influenced by a variety of factors, including plant genotypes [27], growth rate [27], tree age [27, 28], and environmental influences [29, 30]. However, the primary environmental stressors influencing heartwood production in sandal trees have been identified [31]. The variability in the age at which heartwood formation begins in Sandalwood is attributed to the interaction between the genotype and environment [31]. In several parts of India, the percentage of heartwood production increases rapidly from the girth class of 40–50 cm to the girth class of 70–80 cm [32].

A study on the growth performance of sandalwood conducted in the Pyuthan district of Nepal by Poudel et al. [33] revealed that the highest mean annual diameter increment was 51.94 cm, while the lowest was 28.25 cm. The highest mean annual height increment was 6.39 m, and the lowest was 4.47 m. The highest mean annual volume increment was 0.678 m^3^, and the lowest was 0.13 m^3^. The predicted maximum annual income range for *Santalum* album was USD 221-530, accounting for 10%–15% of farmer income, with the minimum range being USD 194-265, or less than 10% of farmer income. The price difference of *Santalum* album between farmers and users in Kathmandu was 2200 times higher [33]. A distinct correlation between sandalwood growth and soil properties has been observed. Soil properties such as pH, water holding capacity, porosity, volume expansion on wetting, exchangeable calcium, magnesium, and available potash positively affect the girth and height increment in sandalwood plants. Genotype-environment interactions may, therefore, explain the variability in the age of onset of heartwood formation reported in *Santalum* album [34].

Issues with high-quality seedlings, technical assistance from associated organizations for disease prevention, general management of farmed sandalwood, and commercialization are the main challenges in sandalwood production in Nepal [9]. In the Pyuthan district, sandalwood is somewhat locally distributed. Additionally, with technical assistance from the Division Forest Office (DFO) in Pyuthan and the sub-DFO in Swargadwari, some regions are developing into centers for sandalwood cultivation. Being a resilient species, Indian Sandalwood can thrive in a range of soil types and is resistant to soil with a pH of up to nine, but it cannot flourish in waterlogged areas. The plant prefers red ferruginous soil that contains gneiss [10].

As an exotic species in Nepal, sandalwood is being cultivated on many private lands and in a few forest areas [9]. It provides good financial returns to private landowners along with their agricultural crops. In the Pyuthan district, the cultivation of sandalwood is considerable, and the DFO Pyuthan provides continuous support to local cultivators by supplying seedlings and other technical assistance. However, very limited studies have been conducted on important aspects of sandalwood cultivation, such as growth performance and soil preference, particularly, in the Pyuthan district. This has resulted in limited knowledge on additional needed interventions for the cultivation and management of sandalwood, and growth performance of the plant as a reference for the appropriate age and size for harvesting in the Pyuthan district and the study area in particular.

In this context, this research focused on the growth performance of sandalwood in areas with particular soil characteristics. A significant amount of money has been invested by the Forest Office in providing seedlings to local farmers, but the actual growth performance in that specific locality has not been assessed. Therefore, the results of this study will be crucial for the future cultivation of sandalwood and can serve as a guideline for determining the most profitable size for harvesting in terms of both time and money.

## 2. Materials and Methods

### 2.1. Study Area

**Figure 1:**
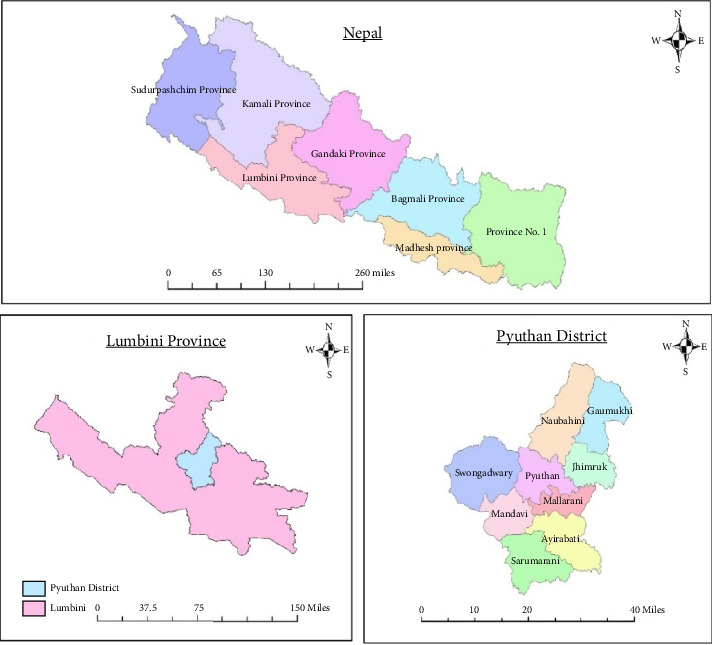
Map showing the study area.

The study was carried out in the Pyuthan district (Figure 1). The Pyuthan district is situated in mid-western part of country, within Lumbini Province. It lies 250 km distance from Kathmandu. It lies in 28.1017° north latitude and 82.8533° longitude. The elevation range of this district from 305 m from the mean sea level to 3659 m. There are altogether nine local governments in the district [9].

In the Pyuthan district, Sandalwood plant has been cultivated in private plantation since more than 20 years [33]. Within the district, Swargadwari municipality, Ward No. 3 was selected for the study as this area is growing as the hub for Sandalwood cultivation in the Pyuthan district as per DFO staff. In particular, four villages (Padampur, Takuratol, Daderi, and Danjanwang) with about 270 households were selected as they cover the highest percentage of the total number of Sandalwood plants.

### 2.2. Data Collection

To document the growth performance of sandalwood in terms of height, diameter, and volume increment, the following methods were used.

#### 2.2.1. Sampling

Purposive sampling followed by random sampling with altogether 80 trees from four hamlets.

#### 2.2.2. DBH and Height Measurement

For this purpose, altogether 80 trees (10 of each size) were measured of 9–16 years. The diameter of the tree was measured by using a diameter tape, and the height was measured with Abney's level. The age of plants was recorded from the year of plantation or sowing by asking the land owner.

To analyze the soil characteristics under Sandalwood plantation in the study area, the following methods were used.

#### 2.2.3. Sampling

Purposive sampling with altogether 12 plants.

For this purpose, 12 (4 ∗ 3) different soil samples were collected from the root zone of seeding, sapling, and tree size plant.

#### 2.2.4. Soil Coring

The soil sample was collected in the field for further examination of its physical (soil color, texture, soil porosity) and chemical (soil organic carbon, nitrogen, potassium, phosphorus, and PH value) properties. For this purpose, 12 (4 ∗ 3) different soil samples were collected from the root zone of seeding, sapling, tree from all four different plots of each village.

To assess the conservation and management problem of Sandalwood in the study area, the following methods were used.

#### 2.2.5. Direct Observation

Trees sampled for Objectives 1 and 2 were also screened to identify insect and disease attack if any, and the data were recorded.

#### 2.2.6. Focus Group Discussion (FGD)

Eight FGDs were conducted to collect data on conservation management problem. For conducting FGD, a checklist was prepared for open discussion on those topics and response was noted down. Severity of each problem was also discussed and recorded.

#### 2.2.7. Key Informant Interview (KII)

Twelve KII was conducted for the triangulation of the information collected from FGD, overall trade trend of sandalwood, and current and needed support from DFO and sub-DFO.

### 2.3. Data Analysis

The mean height and mean annual height increment were calculated by using the following formula:

(1) $$\begin{array}{c} \text{Mean height}\left(\text{MH}\right)=\frac{\text{Sum of total height}}{\text{Total number of tree measured}},\\ \text{MAHI}=\frac{\text{Mean height}}{\text{Age of tree}}. \end{array}$$

The mean diameter and mean annual diameter increment were calculated by using the following formula:

(2) $$\begin{array}{c} \text{Mean diameter}\left(\text{MD}\right)=\frac{\text{Sum of total diameter}}{\text{Total number of tree measured}},\\ \text{MADI}=\frac{\text{Mean diameter}}{\text{Age of tree}}. \end{array}$$

The mean volume and mean annual volume increment was calculated by using the following formula [33]:

(3) $$\begin{array}{c} \text{Mean volume}\left(\text{MV}\right)=\frac{\text{Sum of total volume}}{\text{Total number of tree measured}},\\ \text{MAVI}=\frac{\text{Mean volume}}{\text{Age of tree}}. \end{array}$$

For this purpose, the volume of the tree was calculated using the following formula mentioned in Ref. [35]:

(4) $$\begin{array}{c} \text{Volume}=\frac{\left(3.14d^{2}/4*\text{height}*\text{FF}\right)}{1000}. \end{array}$$

#### 2.3.1. Soil Texture

The particle size analysis method was adopted to determine the proportion of various size particles in soil

(5) $$\begin{array}{c} \text{Soil porosity}=\left(1-\left(\frac{\text{Bulk density}}{\text{particle density}}\right)\right)*100. \end{array}$$

For this, the bulk density and particle density were calculated as

(6) $$\begin{array}{c} \text{Bulk density}=\frac{\text{Mass of the dry soil}}{\text{ Volume of the dry soil}},\\ \text{Particle density}=\frac{\text{Dry mass}\left(g\right)\text{ of soil}}{\text{Volume of soil particles}}. \end{array}$$

#### 2.3.2. The following Chemical Properties Were Assessed

The dry combustion method was used to analyze the carbon content in soil. Nitrogen was analyzed using the Kjeldahl method [36]. Potassium was determined by using the potassium, ammonium acetate method [37]. For phosphorous analysis, Olsen's method was used [38]. The electrometric method was used for pH analysis. The result obtained from soil sample testing was summarized, averaged, and presented in the tabular form for physical and chemical properties separately.

## 3. Results and Discussion

### 3.1. Height Increment

**Figure 2:**
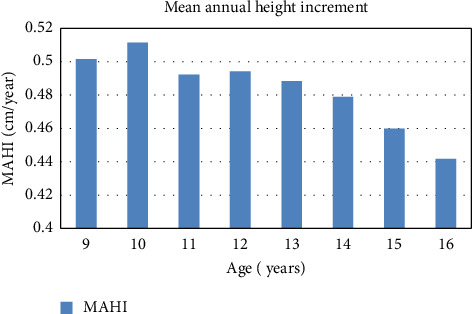
Mean annual height increment.

This result shows the maximum mean annual height increment at the age of 10 years with value 0.5115 ± 0.1123 m/year. The minimum mean annual height increment was found to be 0.4418 ± 0.1680 at the age of 16. Here, the annual rate of height growth is found to increase up to 10 years, and then, the rate is decreasing gradually with the age (Figure 2).

A similar study done in Kwadi of the Pyuthan district shows a considerably different result where the estimated highest mean annual height increment was 6.312 ± 0.07 at the age of 15 years, while it was the lowest at the age of 9 years, i.e., 4.575 ± 0.0064 .

In another site, Bijayanagar, the estimated highest mean annual height increment was 6.39 ± 0.089 m at the age of 15 years, while it was the lowest at the age of 9 years, i.e., 4.5 ± 0.043.

In the next site Ramdi, the highest mean annual height increment was 6.5 ± 0.09 at the age of 15 years, while it was the lowest at the age of 9 years, i.e., 4.47 ± 0.053 mb [33]. The results of these studies are different than my study result.

### 3.2. Diameter Increment

**Figure 3:**
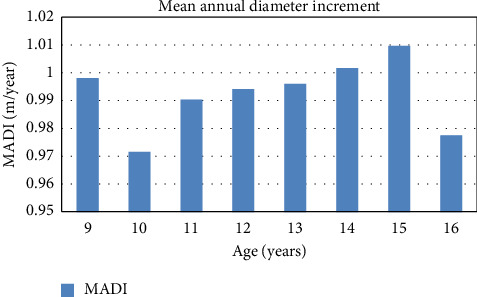
Mean annual diameter increment.

Here, the maximum mean annual diameter increment was at the age of 15 with value 1.0095 ± 0.1019 cm/year. Similarly, the minimum mean annual diameter increment was found at the age of 10 with value 0.9713 ± 0.2188 cm/year. From the age of 10 years, there is a gradual increase in the mean annual diameter increment up to 15 years, and it is found decreased from 15 to 16 years (Figure 3).

A similar study done in another site, i.e., Kwadi of the Pyuthan district, shows that the highest mean annual diameter increment was 47.84 ± 1.720 cm at the age of 16 years while it was minimum at the age of 9 years, i.e., 28.25 ± 0.391 cm.

At another site, i.e., Bijayanagar of the same district, the highest mean annual diameter increment was observed at the age of 16 with 51.94 ± 0.73 cm value, and it was minimum at the age of 9 years with value 28.25 ± 0.391 cm. In Ramdi, the highest mean annual diameter increment was observed at the age of 16 with 50.64 ± 0.94 cm value, and it was minimum at the age of 9 years with value 28.53 ± 0.94 cm [33].

### 3.3. Volume Increment

**Figure 4:**
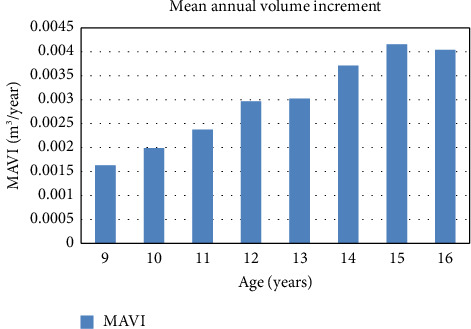
Mean annual volume increment.

This result shows a maximum mean annual volume increment at the age of 15, i.e., 0.004142 ± 0.001941 m^3^/year. The minimum volume increment was found to be 0.001618 ± 0.000431 m^3^/year at the age of 9 (Figure 4).

A similar study done in another site of the Pyuthan district shows a considerably different result. At Kwadi, the highest mean annual volume increment was 0.573 ± 0.018 m^3^ at the age of 16 years, while it was the lowest at the age of 9 years with value 0.144 ± 0.005 m^3^ [33].

At Bijayanagar, the highest mean annual volume increment was 0.678 ± 0.021 m^3^ at the age of 16 years, while the lowest 0.134 ± 0.003 m^3^ at the age of 9 years [33].

At Ramdi, the highest mean annual volume increment was 0.647 ± 0.028 m^3^ at 16 years and the lowest 0.145 ± 0.011 m^3^ at the age of 9 years. In all parameter, the result is different.

### 3.4. Soil Characteristics

The test of major soil characteristics was done to show the exact soil condition of the study area, and the result was found as follows.

#### 3.4.1. Physical Properties

Soil texture and soil porosity were assessed, as they highly effect on the soil aeration, internal drainage, and rooting system of the plant. The texture of the collected soil sample was silty clay-silty clay loam. The soil porosity or the amount of pore space in soil was found ranging from 38.65 to 46.53. On average, 42.56 porosity was concluded.

#### 3.4.2. Chemical Properties

Chemical properties assessed were organic carbon, nitrogen, potassium, and phosphorus contents, and soil pH. The result shows the organic carbon content in the soil sample was 1.42%–2.61%. Nitrogen, phosphorus, and potassium contents were found to be 0.09%–0.182%, 146.74–180.06 kg/ha., and 645.12–1075.2 kg/ha, respectively. Soil pH in soil was found ranging from 7.3 to 8.1.

### 3.5. Conservation and Management Problems in Sandalwood

The cultivation of Sandalwood in the study area is giving a good return to the farmers, and the site is a growing Sandalwood hub in the Pyuthan district; however, some problems are being faced by farmers for its conservation and management. Major problems identified by KIIs, FGD, and field observation can be described as follows.

#### 3.5.1. Conservation Problems

i. Difficulty in saving it in initial 2–4 years: This is most prominent of all. The plant is completely parasitic at this stage of life. So it is very difficult to save it till 2–4 initial years. This is because it needs a proper selection of the host plant, and once it gets established, it starts to grow well. Due to this, extra expenditure is needed for additional number of plants and more time is spent for a single plant establishment. This finally results in reduced net worth of farmers.
ii. Problem of stem decay due to improper pruning: As per the local people, this has resulted in the death of the plant in some cases. In the Sandalwood plant, if pruning is done very close to the main stem, it causes the gradual decay of main stem below that point. As its lower part is decayed, ultimately the plant dies. Although it is a serious, issue very limited cases are observed as farmers are more aware about the economic loss.
iii. Theft: It was a major problem in past years than the present time. There were few Sandalwood plants in the area, and as it gives a good cashback, theft was high. Another reason was the distance of the plant from owner's house and no reach of the owner in night time. Theft of the standing tree mainly during night time for its illegal trade to different parts of the country was common in the past than the present as per the farmers. This resulted in a huge economic loss of farmers. That resulted in the plantation of Sandalwood even in the home garden, nearby area. Consequently, a very large number of plants can be seen in the village and people are more aware about the theft and they are more organized against the illegal theft and export. Many conservation interventions are adopted by farmers. This has an ultimate impact on increasing conservation and production and good economic returns to the farmers.
iv. Leaf eaters and other insects: Leaves of the plant get partially or almost completely destroyed by the leaf eater. However, it has limited impact on the plant as it gets recovered as the larva completes the life cycle. A very limited impact can be observed on the photosynthesis and growth rate of the plant. Moreover, ants and termite also cause damage to the stem, reducing the amount of quality wood.

#### 3.5.2. Management Problems

i. Management of appropriate host: Management of the appropriate host plant throughout the plant life is somehow challenging. It is because most of the plants are grown around the crop field, and a seasonal change in the availability of the host plant causes different nutrient availability to the plant with season. A good growth performance is observed in association with host such as Asuro, ipil-ipil, Mass, Bhatmass, and other legume plants. Managing the availability of these hosts with appropriate care gives a good growth performance as per the farmers. This causes variable growth performance with the location of the individual plant even within the same locality.
ii. Slow growth rate in the transplanted plant as compared to the plant grown in the place of original regeneration: As per most of the farmer experience, a slow growth rate was observed in transplants as compared to the plant naturally grown on site. This is due to their adaptation capacity and rooting strength. This results in the longer growth period to reach the exploitable size than in naturally grown in situ.

### 3.6. Water Availability

Water availability directly influences the growth performance of the plant as water is essential for the metabolic as well as physiological activity of plant. However, the impact is very minimal than the above-mentioned causes.

## 4. Discussion

### 4.1. Mean Annual Height Increment

In this study, the maximum mean annual height increment is 0.5115 ± 0.112311571 m/year, and the minimum mean annual height increment is 0.441875 ± 0.168027775 at the age of 10 and 16 years, respectively. A similar study done in Kwadi of the Pyuthan district shows a considerably different result where the estimated highest mean annual height increment was 6.312 ± 0.07 at the age of 15 years, while it was the lowest at the age of 9 years, i.e., 4.575 ± 0.0064 . In another site, Bijayanagar, the estimated highest mean annual height increment was 6.39 ± 0.089 m at the age of 15 years, while it was lowest at the age of 9 years, i.e., 4.5 ± 0.043. In the next site, Ramdi, the highest mean annual height increment was 6.5 ± 0.09 at the age of 15 years, while it was the lowest at the age of 9 years, i.e., 4.47 ± 0.053 mb [33]. The results of these studies are different than the present study result. The main reason behind this difference in the result might be the difference in the method of result presentation or the site selected for the study.

### 4.2. Mean Annual Diameter Increment

In this study, the maximum mean annual diameter increment is 1.00 ± 0.10 cm/year. Similarly, the minimum diameter increment is 0.97 ± 0.21 cm/year at the age of 15 and 10 years, respectively. A similar study done in another site, i.e., Kwadi of the Pyuthan district, shows a considerably different result as the mean annual diameter increment was the highest, i.e., 47.84 ± 1.720 cm at the age of 16 years, while it was minimum at the age of 9 years, i.e., 28.25 ± 0.391 cm. At another site, i.e., Bijayanagar of the same district, the highest mean annual diameter increment was observed at the age of 16 with 51.94 ± 0.73 cm value, and it was minimum at the age of 9 years with value 28.25 ± 0.391 cm. In Ramdi, the highest mean annual diameter increment was observed at the age of 16 with 50.64 ± 0.94 cm value, and it was minimum at the age of 9 years with value 28.53 ± 0.94 cm [33]. The results of these studies are different than the present study result, which might be due to the difference in the method of the result presentation site selected for the study.

### 4.3. Mean Annual Volume Increment

In this study, the maximum mean annual volume increment is 0.004 ± 0.001 m^3^/year and the minimum volume increment is 0.001 ± 0.0004 m^3^/year at the age of 15 and 9 years, respectively.

At Kwadi, the highest mean annual volume increment was 0.573 ± 0.018 m^3^ at the age of 16 years, while it was the lowest at the age of 9 years with value 0.144 ± 0.005 m^3^ [33]. At Bijayanagar, the highest mean annual volume increment was 0.678 ± 0.021 m^3^ at the age of 16 years, while the lowest 0.13 ± 0.003 m^3^ at the age of 9 years [33]. At Ramdi, the highest mean annual volume increment was 0.64 ± 0.02 m^3^ at 16 years and lowest 0.14 ± 0.01 m^3^ at the age of 9 years. The results of these studies are different than the present study result, which might be due to the difference in the method of result presentation or the site selected for the study.

In all parameters, the result is different in the study area and other reference sites. This might be due to the variation in site characteristics or the method of result presentation.

### 4.4. Soil Properties

In the study area, the texture of the soil sample found silty clay-silty clay loam, soil porosity was found ranging from 38.65 to 46.53, and on average, 42.56 porosity was concluded. Organic carbon content, nitrogen content, potassium content, and phosphorus content in the soil are found as 1.42%–2.61%, 0.09%–0.182%, 645.12–1075.2 kg/ha, and 146.74–168.02 kg/ha, respectively, and soil pH ranges from 7.3 to 8.1. Other study results show that the Sandalwood plant can grow in any well-drained soil having good organic matter; however, red sandy loam soil is best for their growth and yield. Sandalwood grows best in slightly alkaline soil with pH range 6.5–7.5 (https://www.agrifarming.in).

Another study shows that Sandalwood is suitable to grow in a variety of soil, including sandy soils, red clay soils, and clay-rich black soils that can adjust to rocky hard ground soil types; however, the pH level is 6–7.5 [39].

### 4.5. Conservation and Management Problems

Difficulty to save it in initial 2–4 years as well as the water availability problem is major of all problems as the plant is completely parasitic at this stage. This causes additional cost for the establishment of plant. Young Sandalwood should be planted into pits at the start of the rainy season as it does not tolerate drought. The Sandalwood plant and host are planted mostly in alternate or adjacent pits and occasionally in the same pits [16].

Another problem faced is the management of appropriate host throughout its life because of the seasonal change in the availability of the host plant in the agricultural land. This causes variable growth performance with the location of the individual plant even within the same locality. A slow growth rate in the transplanted plant due to their adaptation capacity and rooting strength is also a problem. This results in the longer growth period to reach the exploitable size than in naturally grown in situ. Sandalwood will grow best in the cultivated land with host plants already established to provide shade [16, 40].

The study shows better growth of Sandalwood can be observed with mango (*Mangifera indica*) under intensively managed plantation, followed by Amla (*Phyllanthus emblica*) [41, 42]. Greater height, collar diameter, crown size, clear bole, and survival of Sandal with (*Citrus aurantium*) are seen compared to *Casuarina equisetifolia* and *Punica granatum* [41, 43].

Decay due to inappropriate pruning has caused economic loss. The wound caused by severe tissue connection and enhance the infection of decay fungi. It attracts the infestation of insect pests, mainly the stem and wood borers. The wounds caused by pruning sever tissue connections and enhance the infection of decay fungi. It attracts the infestation of insect pests, particularly, the stem and wood borers [44]. For this, pruning from very close to the bole should be avoided.

Theft is another problem in the study area; however, the case is minimized as compared to the past years. Few Sandalwood plants in the area, the distance of the plant from owner's house, and no reach of the owner in night time and a good cashback are the major causes of the problem. It caused huge economic loss in past that resulted in the plantation of Sandalwood in mass, adoption of more conservation interventions, and a good impact on conservation and production and good economic returns to the farmers. The use of protective mess in many public and private lands from being theft is common in India [45]. A study in India shows challenge in the physical protection of the mature tree mainly from theft and managing the best host plant and challenge to maintain sustained income to meet the protection and maintenance of the plant [10]. The problem of theft is also found in the study area, whereas there is no worry about the sustained income during the growth period of the plant as most of the plants are grown in the boarder of the cultivated land and farmers have other different sources of regular income such as farming, animal husbandry, business, teaching, and daily wages-based works on the study area in contrast to the Indian scenario.

Leaf eater causes a negative impact on the photosynthesis and growth rate of the plant by reducing the leaf surface area exposed. Moreover, ants and termite also cause damage to the stem, reducing the amount of quality wood. Out of more than 150 insects known to infest Sandalwood, a few have been recorded as serious huge economic losses. Defoliators, sapsuckers, stem borers, and termites are the most important of all. Hence, to raise a healthy plantation, knowledge on its diseases and insect pests is of utmost importance [46]. Repeated defoliation may result in death. Therefore, physical removal, pesticide treatment, or flame treatment are recommended by targeting the colony [47]. There is water availability problem in dry season, which is supposed to cause heat stress and impact on the plant metabolic activity.

The successful cultivation of Indian Sandalwood (*Santalum album*) in non-native regions such as Australia and Fiji provides valuable insights that can be applied to similar efforts in Nepal. In Australia, the establishment of the world's largest plantation sandalwood supply is supported by well-drained soils, sophisticated irrigation systems, and effective pest management practices [13]. These strategies are essential for overcoming the challenges posed by Australia's temperate climate, which requires supplemental irrigation to ensure consistent moisture levels. The use of compatible host species and integrated pest management (IPM) further enhances the growth and productivity of sandalwood in this region [13].

In contrast, Fiji benefits from a semitropical climate with adequate rainfall, reducing the need for extensive irrigation systems [14]. The well-drained soils and favorable climatic conditions in Fiji support the natural growth of sandalwood. Additionally, the promotion of agroforestry practices, which integrate sandalwood cultivation with other crops, enhances productivity, and provides economic benefits to local farmers [48]. Fiji's approach to the domestication and breeding of sandalwood aims to establish a sustainable industry, offering valuable lessons for Nepal.

A comparative analysis of these regions highlights the importance of soil type, rainfall, temperature, and management strategies in the successful cultivation of Indian Sandalwood. Australia's reliance on irrigation systems contrasts with Fiji's natural rainfall advantage, demonstrating different approaches to managing moisture levels. The selection of compatible host species and implementation of IPM practices are critical factors in both regions, ensuring the health and productivity of sandalwood trees.

The silvicultural system for Indian Sandalwood *(Santalum album)* involves several critical practices to ensure optimal growth and productivity. First, selecting well-drained soils such as sandy loam and clay loam is essential, as these provide the necessary nutrients and moisture retention for sandalwood [13]. Given its hemiparasitic nature, sandalwood requires a compatible host plant like Acacia, Eucalyptus, or Casuarina to obtain essential nutrients, making host plant selection crucial [14]. Proper planting density and spacing, typically around 2.5 × 2.5 m, help reduce competition for resources and ensure adequate light penetration [48]. In regions with lower rainfall, such as parts of Australia, sophisticated irrigation systems, particularly drip irrigation, are employed to maintain consistent moisture levels [15]. IPM practices are vital to protect the trees from diseases and pests, ensuring healthy growth. Regular pruning and thinning also contribute to the health and productivity of sandalwood by removing dead or diseased branches and reducing competition. In Fiji, agroforestry practices, which integrate sandalwood with other crops, enhance productivity, and offer economic benefits to local farmers, demonstrating a model that could be adapted for Nepal [14]. By adopting these silvicultural practices, the growth and productivity of Indian Sandalwood can be optimized, providing a sustainable and profitable cultivation system that can be tailored to the specific ecological and socioeconomic conditions of regions like Nepal.

For Nepal, these international experiences offer practical insights into policy development, resource management, and farmer engagement. By adopting sophisticated irrigation techniques and IPM practices from Australia, and agroforestry practices from Fiji, Nepal can develop a robust silvicultural package tailored to its specific ecological and socioeconomic conditions. Understanding the regulatory frameworks and market strategies employed by these countries can help Nepal create policies that support the cultivation and trade of sandalwood, ensuring economic benefits for local communities while preserving ecological balance [15, 48].

## 5. Conclusion

The study shows that the mean annual height increment progressively increases at the early stage of Sandalwood plant life up to 10 years (0.51 m/year), and then, it decreases gradually and is minimum at the age of 16 years (0.44 m/year). On other hand, the mean annual diameter increment is progressively decreasing up to the age of 10 years, and then, it starts to increase with age up to a certain age limit.

In this study, the highest mean annual diameter increment was at the age of 15 years (1.009 cm/year) and the lowest at the age of 10 years (0.97 cm/years). From this result, it can be concluded that there is a slower increment in the diameter, while the height is increasing faster in early age up to 10 years and then, the diameter starts increasing faster and the height increment becomes slower with age. The highest mean annual volume increment was found at the age of 15 years (0.004142 m^3^/year), and the lowest mean annual volume increment was at the age of 9 years (0.001618 m^3^/year). This shows the rate of volume increment is progressively increasing with age up to a certain age limit, giving an increasing trend of mean volume increment with age. Further studies on the variation in the seed diameter and its effect on nursery and field growth are recommended.

This type of growth performance of the Sandalwood plant was observed in that particular soil with soil texture ranging from silty clay to silty clay loam and soil porosity 38.65–46.53 with average value 42.56 having soil pH range 7.3–8.1. Soil organic carbon was found to be 1.42%–2.61%, nitrogen 0.09%–0.18%, phosphorus 146.74–180.60 kg/ha, and potassium 645.12–1075.20 kg/ha.

Difficulty to save it in initial 2–4 years age, theft, decay due to improper pruning, and leaf eaters and ants and termite attack are major conservation problems. However, a slow growth rate in the transplanted plant, the management of appropriate host, and water availability are the major management problems found in the study area.

## Data Availability

The data that support the findings of this study are available from the corresponding author upon reasonable request.
